# Targeting Inflammatory Pathways in Atherosclerosis: Exploring New Opportunities for Treatment

**DOI:** 10.1007/s11883-024-01241-3

**Published:** 2024-10-15

**Authors:** Alessia d’Aiello, Simone Filomia, Mattia Brecciaroli, Tommaso Sanna, Daniela Pedicino, Giovanna Liuzzo

**Affiliations:** 1 Department of Cardiovascular Sciences- CUORE, Fondazione Policlinico Universitario A. Gemelli - IRCCS, Largo A. Gemelli 8, 00168 Rome, Italy; 2 Department of Cardiovascular and Pulmonary Sciences, Catholic University School of Medicine, Largo F. Vito 1, 00168 Rome, Italy

**Keywords:** Atherosclerosis, Inflammation, Colchicine, Interleukin-1, Interleukin-6, Canakinumab

## Abstract

**Purpose of the Review:**

This review discusses the molecular mechanisms involved in the immuno-pathogenesis of atherosclerosis, the pleiotropic anti-inflammatory effects of approved cardiovascular therapies and the available evidence on immunomodulatory therapies for atherosclerotic cardiovascular disease (ACVD). We highlight the importance of clinical and translational research in identifying molecular mechanisms and discovering new therapeutic targets.

**Recent Findings:**

The CANTOS (Canakinumab Anti-Inflammatory Thrombosis Outcomes Study) trial was the first to demonstrate a reduction in cardiovascular (CV) risk with anti-inflammatory therapy, irrespective of serum lipid levels.

**Summary:**

ACVD is the leading cause of death worldwide. Although targeting principal risk factors significantly reduces CV risk, residual risk remains unaddressed. The immunological mechanisms underlying atherosclerosis represent attractive therapeutic targets. Several commonly used and non-primarily anti-inflammatory drugs (i.e. SGLT2i, and PCSK9i) exhibit pleiotropic properties. Otherwise, recent trials have investigated the blockade of primarily inflammatory compounds, trying to lower the residual risk via low-dose IL-2, PTPN22 and CD31 pathway modulation. In the era of precision medicine, modern approaches may explore new pharmacological targets, identify new markers of vascular inflammation, and evaluate therapeutic responses.

## Introduction

Atherosclerosis is the leading cause of major cardiovascular events. Inflammatory mechanisms, involving both the innate and adaptive immune systems, play a crucial role in the initiation and progression of the atherosclerotic plaques. Several commonly used clinical drugs exhibit pleiotropic anti-inflammatory properties. Conversely, numerous trials have investigated the blockade of specific inflammatory pathways, often yielding conflicting or inconclusive results. This review aims to provide an updated overview of current scientific evidence on anti-inflammatory and immunomodulatory therapies targeting atherosclerosis and to evaluate their future directions.

### Immune Pathogenesis of Atherosclerosis

Atherosclerosis is a chronic inflammatory disease characterized by the activation of both the innate and adaptive immune systems [1, 2]. Currently, available therapies for the prevention and treatment of atherosclerotic cardiovascular diseases (ACVD) primarily focus on managing modifiable risk factors, i.e. dyslipidemia, and hypertension, and inhibiting platelets aggregation and thrombosis. However, this approach still leaves a significant number of patients at high and persistent risk of acute events.

In this context, targeting the underlying immunological mechanisms of atherosclerosis appears very attractive to further reduce the burden of ACVD [3, 4]. It is crucial to recognize that certain immunological mechanisms protect against atherothrombotic events. This underscores the importance of targeting the effectors of atherosclerosis while preserving protective mechanisms, thereby emphasizing the need for precision medicine [5–7].

**Fig. 1:**
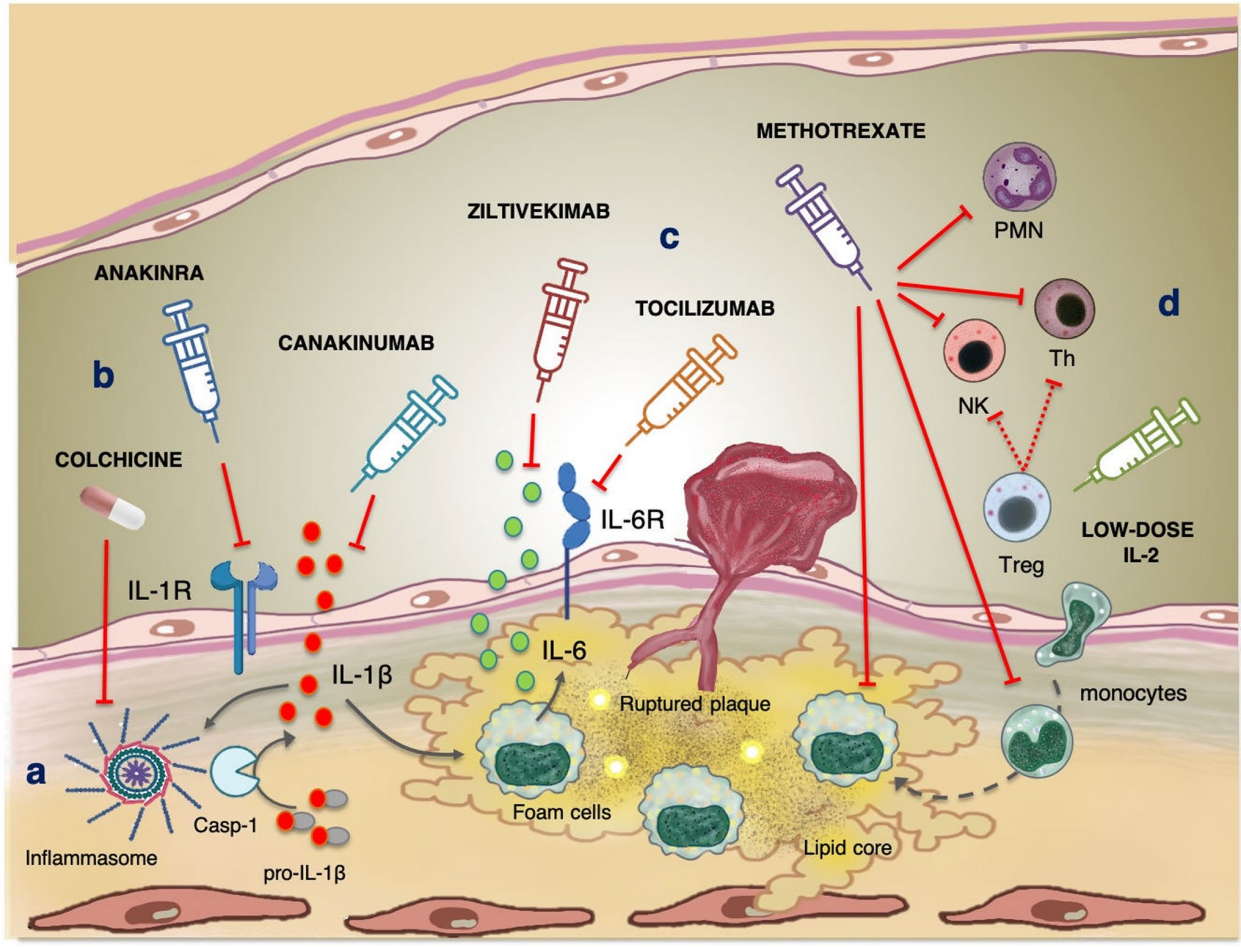
Anti-inflammatory drugs and atherosclerosis (**a**) Inflammasome activation leads to caspase-1 (Casp-1) activation and cleavage of pro-interleukin 1β (pro-IL-1β) to the active IL-1β form. IL-1, in turn activates inflammasome and stimulate the release of IL-6 (**b**) While colchicine exerts indirect effects leading to inflammasome blockade, anakinra and canakinumab interfere with the IL-1β pathways by blocking the IL-1 receptor (IL-1R) and IL-1β, respectively (**c**) IL-6 can bind to the membrane-bound IL-6 receptor (IL-6R) present on hepatocytes, some leucocytes, and endothelial cells; ziltivekimab is a IL-6 ligand monoclonal antibody, while tocilizumab acts on IL-6R, both inhibiting IL-6 pathway (**d**) By inducing adenosine monophosphate (AMP) deaminase inhibition in several immune cells [e.g., natural killer cells (NK), T cells (Th), macrophages, monocytes, and neutrophils (PMN)], methotrexate leads to increased levels of AMP and adenosine, which in turn are responsible for its anti-inflammatory effects. Low-dose IL-2 therapy predominantly activates T regulatory cells (Treg) that express high level of IL-2 receptor, promoting immunotolerance

The involvement of the immune system in the pathogenesis of atherosclerosis has been recognized for decades (Fig. 1). The accumulation of modified lipoproteins, such as ox-LDL, in the sub-endothelium activates damage-associated molecular patterns (DAMPs) receptors on various cell types, causing endothelial dysfunction. This triggers the expression of adhesion molecules (VCAM-1, E-selectin, P-selectin) and the secretion of pro-inflammatory cytokines and chemokines, which recruit mononuclear cells to the vessel intima. Within the plaque, these cells differentiate into dendritic cells and macrophages, which internalize ox-LDL through scavenger receptors, forming the so-called “foam cells”. Foam cells produce large amounts of inflammatory cytokines (IL-1β, TNF, IL-6) and undergo cell necrosis, forming the necrotic core of the plaque and increasing local inflammation. Dendritic cells, as antigen-presenting cells (APCs), bridge innate and adaptive immunity by activating B and T lymphocytes [8, 9]. In patients presenting with acute coronary syndromes (ACS) and plaque rupture [10], previous evidence proved a significant expansion of aggressive T lymphocytes without a corresponding activation of regulatory T cells (Treg cells) deputed to control the adaptive immune response. Inflammatory mediators also induce migration and proliferation of smooth muscle cells (VSMCs) in the tunica media, contributing to plaque stabilization through a fibro-proliferative response. Furthermore, some VSMCs adopt macrophage-like properties, further contributing to the necrotic core formation. Inflammatory response triggers the production of proteases, including metalloproteinases, which degrade the extracellular matrix, ultimately leading to plaque rupture, thrombosis, ischemia, and necrosis [11].

From the molecular perspective, the main inflammatory pathway identified as a therapeutic target in atherosclerosis is the NLRP3 (Nod-Like Receptor Pyrin Domain-Containing Protein 3) inflammasome. It is activated by DAMPs and directly by crystallized ox-LDL, leading to caspase-1 activation and cleavage of proIL-1β and proIL-18 into their active forms. This process triggers IL-6 secretion and, downstream, the production of C-reactive protein (CRP) by hepatocytes [12, 13]. Elevated expression of NLRP3 and IL-1β has been observed in circulating monocytes and epicardial adipose tissue of ACS patients [14]. High-sensitivity CRP (hs-CRP) is a useful marker of systemic inflammation. Its prognostic value has been recognized in acute coronary syndromes (ACS), as well as among asymptomatic individuals, since nineties [15] and, in clinical practice, hs-CRP measurement is more convenient than other cytokines (IL-1, IL-6), due to its greater stability [16].

Recent evidence provides new interesting insights into atherosclerosis pathogenesis. Indeed, the gut microbiota, producing multiple molecules with pleiotropic effects, mediates the relationship between lifestyle and dietary factors, systemic chronic inflammation and the progression of atherosclerosis [17]. Additionally, clonal hematopoiesis of indeterminate potential (CHIP), may be involved in this complex scenario and has been proposed as a cardiovascular disease (CVD) risk factor [18, 19].

Understanding the molecular pathways underlying atherosclerosis inflammatory features may pave the way for significant advancements in treating ACVD, bringing us closer to achieving personalized medicine. Table 1 summarizes the major randomized clinical trials (RCTs) directly exploring anti-inflammatory therapy in ACVD.

**Table 1:**
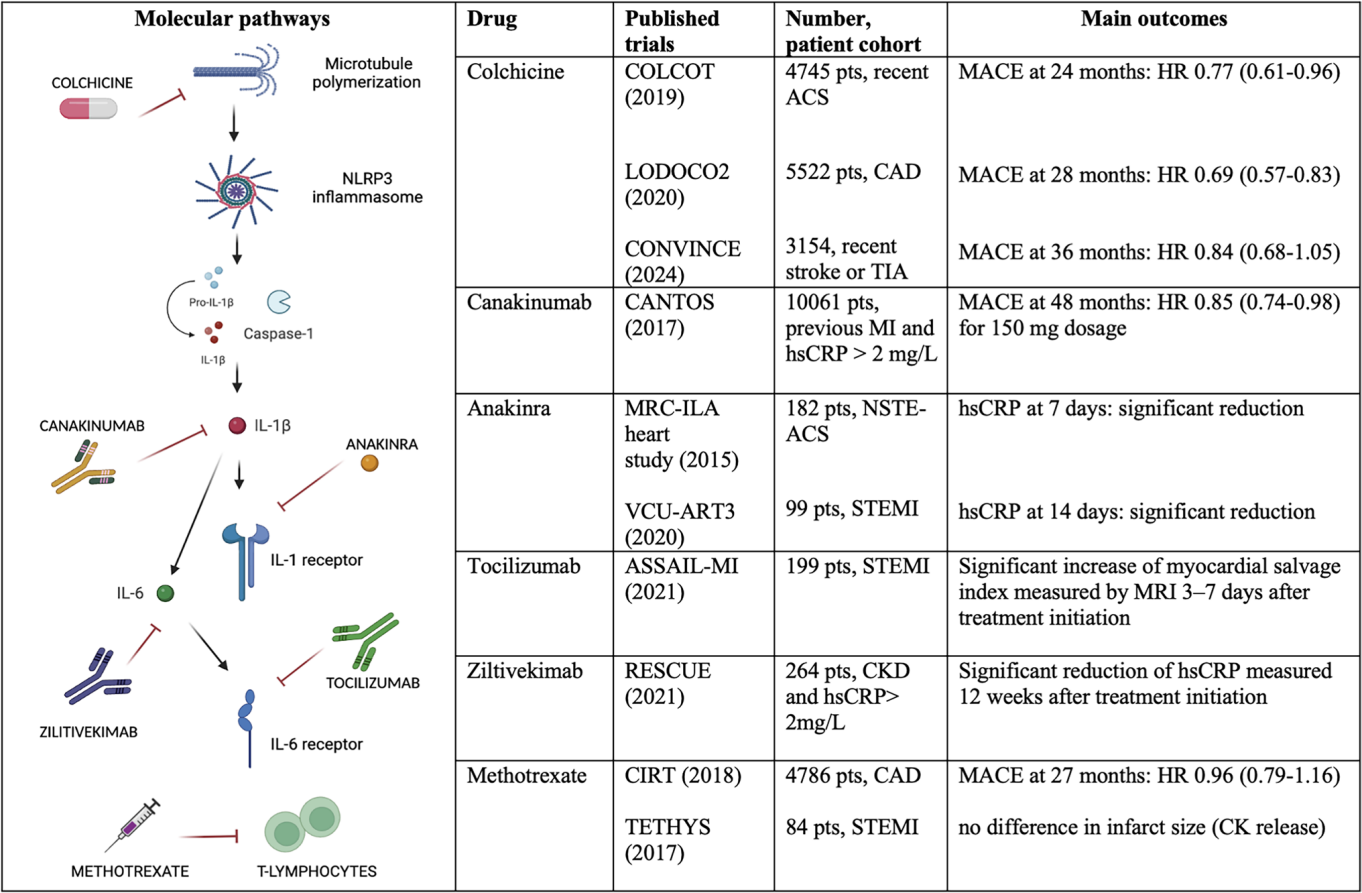
Main phase III RCTs directly exploring anti-inflammatory therapy in ACVD

*ACS* Acute Coronary Syndrome, *ASSAIL-MI* ASSessing the effect of Anti-IL-6 treatment in Myocardial Infarction, *CAD* Coronary Artery Disease, *CANTOS* Canakinumab AntiInflammatory Thrombosis Outcomes Study, *CIRT* Cardiovascular Inflammation Reduction Trial, *CKD* Chronic kidney disease, *COLCOT* Colchicine Cardiovascular Outcomes Trial, *CONVINCE* Colchicine for Prevention of Vascular Inflammation in Non-CardioEmbolic Stroke, *hsCRP* high-sensitivity C-Reactive Protein, *LODOCO2* Low-Dose Colchicine-2 Trial, *MACE* Major Adverse Cardiac Events, *MI* Myocardial Infarction, *MRC-ILA* Effect of IL-1 receptor antagonist theraphy con markers of inflammation in non-ST elevation acute coronary syndromes, *NSTE-ACS* Non-ST Elevation – Acute Coronary Syndrome, *STEMI* ST-Elevation Myocardial Infarctio, *RESCUE* Reduction in Inflammation in Patients with Advanced Chronic Renale Disease Utilizing Antibody Mediated IL-6 Inhibition, *TIA* Transien Ischemic Attack, *TETHYS* The Effects of mETHotrexate Therapy on ST Segment Elevation Myocardial InfarctionS trial, *VCU-ART3* Virginia Commonwealt University-Anakinra Remodeling Trial 3

## Pleiotropic Effects of Non-Primarily Anti-Inflammatory Drugs

### Lipid Lowering Drugs

#### Statins

Statins are HMG-CoA reductase inhibitors able to reduce LDL-C levels. Statins exhibit pleiotropic and anti-inflammatory effects, resulting in a dose-dependent reduction in hs-CRP [20]. The JUPITER primary prevention trial demonstrated that in 17,802 healthy subjects with LDL below 130 mg/dL and hs-CRP above 2 mg/dL, those who were administered 20 mg rosuvastatin experienced a 37% reduction in hs-CRP levels and a significant reduction in adverse cardiovascular events, compared to those receiving placebo [21]. The effect of rosuvastatin in subjects with low cardiovascular risk (non-smokers, non-overweight, LDL < 100 mg/dL) but with high hs-CRP levels was comparable to that in high-risk subjects in terms of risk reduction, highlighting that addressing systemic chronic inflammation provides additional cardiovascular risk reduction, beyond the benefits of lowering LDL levels [22]. Additionally, in a population of 3,745 patients derived from PROVE-IT TIMI 22, the greatest relative risk reduction occurred in patients achieving LDL < 70 mg/dL and hs-CRP < 1 mg/dL[23]. Exploring the reasons beyond CRP level decrease, previous research revealed that statins attenuate T-cell activation and proliferation, inhibit pro-inflammatory cytokine secretion and enhance anti-inflammatory cytokine secretion. Interestingly, a prior study from our group showed that in ACS, ex-vivo atorvastatin treatment decreases the expression of transcription factors in T-cells, i.e. the “immediate-early response protein” EGR1, resulting in inhibition of pro-inflammatory effector T-cells and activation of regulatory T-cells. EGR1 reduction by atorvastatin has also been confirmed in-vivo, after a single high-dose treatment, suggesting the role of early initiation of statin therapy after ACS [24].

#### PCSK9i

PCSK9 inhibitors (PCSK9i), such as evolocumab and alirocumab, increase LDL receptor availability by preventing its degradation, leading to consistent reductions in LDL-C levels. In vitro studies and animal models suggest that PCSK9i also have pleiotropic and anti-inflammatory effects, i.e. reducing macrophage recruitment and cytokine production [25]. Furthermore, PCSK9i may improve plaque morphology, reducing its lipid content, and decreasing monocyte migratory capacity at the subendothelial level, independently of circulating cholesterol levels [26].

However, the anti-inflammatory effects in humans are controversial. Some meta-analyses and original studies documented no effect of PCSK9i treatment on hs-CRP levels [27, 28]. In contrast, some imaging studies with 18FDG-PET and histological samples from patients undergoing carotid endarterectomy suggest that PCSK9i might exert anti-inflammatory effects on the vessel wall by acting on the NLRP3 inflammasome pathway, without affecting circulating biomarkers [29, 30].

Recently, the PACMAN-AMI (Effects of the PCSK9 Antibody Alirocumab on Coronary Atherosclerosis in Patients with Acute Myocardial Infarction) trial proved that, among patients with AMI, the addition of subcutaneous biweekly alirocumab, compared with placebo, to high-intensity statin therapy resulted in significantly greater coronary plaque regression in non–infarct-related arteries after 52 weeks. In particular, in the alirocumab group, the Authors observed a greater reduction in lipid burden by near-infrared spectroscopy (NIRS) and increase in OCT-measured minimal fibrous cap thickness, both hallmarks of vulnerable plaque [31].

## New Anti-Diabetic Drugs

### Sodium-Glucose Cotransporter 2 Inhibitors (SGLT2I) and Glucagon-Like Peptide 1 Receptor Agonists (GLP1-Ra)

In the context of regulatory approval trials, sodium-glucose cotransporter 2 inhibitors (SGLT2i) and glucagon-like peptide 1 receptor agonists (GLP1-Ra) proved a reduced incidence of major adverse cardiovascular events (MACEs). Interestingly, the cardioprotective abilities of these new anti-diabetic drugs seem to run beyond the mere glycemic control. SGLT2i therapy has been associated with a decrease in leptin concentration and lower levels of CRP, tumour necrosis factor-alpha (TNF-α), interleukin-6 (IL-6), and interferon-gamma (IFN-γ) [32]. Experimental data disclosed that empagliflozin may downregulate the NLRP3 inflammasome through the AMPK pathway. In particular, SGLT2i enhance the plasma concentration of ß-hydroxybutyrate, a potent NLRP3 inflammasome blocker [33].

In the last years, GLP1-Ra raised great interest because of the hypothesis of cardioprotection in the setting of ischemia–reperfusion damage. Additionally, GLP1-Ra significantly limit vascular monocyte adhesion and macrophages and metalloproteinases accumulation in the atherosclerotic plaque [34].

A detailed discussion of new anti-diabetic drugs goes beyond the aim of this review, but it is important to highlight that new evidence, i.e. the SELECT (Semaglutide Effects on Cardiovascular Outcomes in People with Overweight or Obesity trial) [35] and the SURMOUNT (Tirzepatide Once Weekly for the Treatment of Obesity) [36] trials represent a major step forward in the development of effective treatment options for obese individuals, irrefutably linking inflammation, obesity and ACVD.

## Other Drugs

Aspirin (acetylsalicylic acid) has both anti-platelet and anti-inflammatory properties. A 1997 study showed that aspirin reduces cardiovascular risk more significantly in patients with high CRP levels, with the reduction being proportional to the decrease in CRP. This result indicates that the benefit extends beyond platelet inhibition alone, which is not affected by CRP levels [16]. Metoprolol, a β1-selective beta-blocker, is widely used in ACS for its effects on cardiomyocytes. A 2017 study demonstrated a reduction in infarct size and incidence of MVO with metoprolol use, due to the inhibition of neutrophils recruitment and their activation mediated by β1-adrenergic receptor blockade [37]. Furthermore, users of renin-angiotensin system inhibitors (RASIs) had lower hs-CRP levels, likely due to the role of RAS blockade in improving immune function [38].

## IL-1 Pathway

Interleukin-1 pathway inhibitors include canakinumab, anakinra, and xilonix. IL-1β inhibition has been the first anti-inflammatory approach to complete the transition from pre-clinical to clinical studies in CVD.

The CANTOS (Anti-inflammatory therapy with canakinumab for atherosclerotic disease) trial, a phase III randomized double-blind placebo-controlled study, investigated the effects of canakinumab, an anti-IL-1β monoclonal antibody, in 10,061 patients with previous myocardial infarction (MI) and hs-CRP > 2 mg/L. In this cornerstone trial, at a dose of 150 mg every three months, canakinumab significantly reduced cardiovascular events compared to placebo (hazard ratio [HR] 0.85, 95% confidence interval [CI] 0.74 to 0.98; *P* = 0.021), irrespective of serum lipid levels. Canakinumab produced a dose-dependent reduction in hs-CRP and IL-6; patients achieving hs-CRP < 2 mg/dL and IL6 < 1.65 ng/L derived the greatest benefit. Canakinumab increased the number of fatal infections and showed no overall mortality benefit (HR 0.94; 95% CI, 0.83 to 1.06; *P* = 0.31) [39]. CANTOS represented the first step in the endorsement of anti-inflammatory therapies on top of intense lipid lowering in the prevention and treatment of ACVD, especially for patients remaining at high risk of acute events after aggressive control of risk factors. Yet, IL-1β inhibition needs to be evaluated against side effects and tolerability, and the selection of patients who can benefit most from this treatment is crucial [40].

Anakinra is an IL-1α and IL-1β receptor antagonist. The MRC-ILA Heart Study demonstrated that anakinra significantly reduces hs-CRP levels in NSTEMI patients but not Troponin T levels, and was associated with an increase in MACE at 12 months [41]. In three studies by Abbate et al. in STEMI patients, anakinra significantly reduced hs-CRP levels and the risk of death, new-onset heart failure, and hospitalization for heart failure [42–44].

Xilonix is an anti-IL1α monoclonal antibody. In a small phase 2 study involving 43 patients with superficial femoral artery (SFA) stenosis undergoing percutaneous revascularization, xilonix therapy showed a non-significant reduction of MACE and SFA restenosis at three months during the drug's infusion period, with no difference from placebo at 12 months [45].

Based on these studies, the inhibition of IL-1 pathway is attractive for both chronic ACVD and ACS, without definite evidence of a prognostic benefit, which may be confirmed with future trials in specific subset of patients.

## Methotrexate

Methotrexate (MTX) is a disease-modifying antirheumatic drug that inhibits dihydrofolate reductase, suppressing T cell activation and proliferation. In observational studies, rheumatoid arthritis (RA) and psoriatic arthritis patients who received MTX had a reduction in cardiovascular events compared to other drugs or placebo [46].

The CIRT trial was therefore conducted to investigate the impact of low-dose methotrexate in 4,786 patients with stable atherosclerosis, with previous MI or multivessel coronary artery disease and either type 2 diabetes or metabolic syndrome. The trial failed to demonstrate a significant reduction in cardiovascular events compared to placebo. No effect was seen on IL-1β, IL-6, and CRP levels. The lack of efficacy of low-dose MTX was attributed to its poor effectiveness in reducing low-grade chronic inflammation characterizing atherosclerosis. Supporting this perspective, CIRT enrolled patients had lower median hs-CRP levels (median value 1.6 mg/dL) compared to RA or psoriatic arthritis patients. Patients treated with MTX had a higher rate of non-basal-cell skin cancer, cytopenia, and liver dysfunction [47].

Additionally, the administration of MTX to patients with STEMI in the TETHYS trial did not provide any benefit and was associated with a worsening of left ventricle ejection fraction (LVEF) at 3 months [48]. The above-mentioned studies have excluded methotrexate from the candidate drugs to reduce residual cardiovascular inflammatory risk.

## Colchicine

Colchicine is a well-known inexpensive anti-inflammatory drug, primarily used in gout and pericarditis. It exerts its effects by binding to tubulin and preventing microtubule polymerization, interfering with leukocyte function and inflammasome formation.

Firstly, the LoDoCo, a single-blinded non-placebo-controlled trial including 532 patients with stable coronary artery disease, demonstrated that low-dose colchicine (0.5 mg/day) was effective in reducing MACE. The reduction in the primary outcome was primarily driven by a decrease in ACS at the 3-year follow-up [49].

In 2019 the results of the COLCOT trial were published: 4,745 patients within 30 days after a MI were randomly assigned to receive either 0.5 mg colchicine or placebo, demonstrating a significant reduction in the incidence of MACE at 2 years in the treated group (HR 0.77; CI 0.61–0.96; *P* = 0.02). Furthermore, significant reductions were observed in stroke and hospitalization for unstable angina, components of the composite primary outcome. The reduction in clinical outcomes did not coincide with a significant parallel decrease in CRP levels compared to placebo, highlighting how colchicine acts through a different inflammatory pathway [50].

In 2020, the LoDoCo2 trial demonstrated the effectiveness of colchicine in reducing cardiovascular events in patients with stable coronary artery disease, using the same dosage of 0.5 mg/die. The trial included 5,522 patients, with a significant reduction of MACE at 28 months of median follow-up (HR 0.69; CI 0.57–0.83; *P* < 0.001). Among the components of the composite primary outcome, there was a significant reduction in MI and ischemia-driven coronary revascularization, with a trend towards reduction also observed for stroke and cardiovascular mortality [51]. There was an alert due to a borderline increase in non-cardiovascular mortality, apparently not explained by a higher incidence of drug-related oncological or infectious diseases. CRP levels were not explored at baseline and during the trial.

The recently published CONVINCE phase-3 trial showed a trend in MACE reduction in patients with non-embolic ischemic stroke treated with colchicine versus placebo, although the results were not statistically significant [52].

The ongoing large CLEAR SYNERGY trial is investigating the efficacy of colchicine and spironolactone in reducing the recurrence of MACE in patients with acute MI treated with PCI [53].

Overall, colchicine has proven to be an effective drug for reducing residual cardiovascular risk, supported by robust and reproducible data. These results led to its inclusion in the European Society of Cardiology (ESC) 2023 guidelines for secondary cardiovascular prevention after ACS with a high level of evidence (Class A) but a low recommendation grade (Class IIa), for patients with uncontrolled risk factors or recurrent events [54]. Contemporarily, the Food and Drug Administration (FDA) approved low-dose colchicine for reducing CV events in patients who already have ASCVD or those at risk of developing it.

## IL 6 Pathway

Tocilizumab is a humanized anti-IL-6R monoclonal antibody used in several inflammatory and autoimmune disorders. In 2016, a small phase-2 trial proved the efficacy of tocilizumab in reducing CRP and Troponin T levels during hospitalization in patients with NSTEMI [55]. The subsequent ASSAIL-MI trial demonstrated an increase in the myocardial salvage index, evaluated with Cardiac Magnetic Resonance (CMR), in 200 patients with STEMI randomized to receive a single dose of intravenous tocilizumab during PCI or placebo [56]. There was also a significant reduction in microvascular obstruction and CRP levels during hospitalization in patients treated with tocilizumab, while the reduction in infarct size was not statistically significant. Interestingly, patients with a door-to-balloon time longer than 3 h experienced the greatest benefit. Tocilizumab seemed to effectively address ischemia–reperfusion injury, among the main contributors to myocardial damage during primary PCI. No major safety issues were outlined in the previously mentioned trials. A following sub-study of the ASSAIL-MI showed that tocilizumab mainly affected neutrophil count and function, without interfering with lymphocyte activity [57].

While trials with tocilizumab have focused on ACS patients, another anti-IL-6 antibody called ziltivekimab is currently under investigation to assess the efficacy of anti-inflammatory therapy in patients at high cardiovascular risk. The phase-2 RESCUE trial initially supported the ziltivekimab's efficacy in reducing inflammatory biomarkers, including CRP, among patients with moderate to severe kidney disease and elevated hs-CRP levels at baseline [58]. A post hoc analysis highlighted a reduction in neutrophil–lymphocyte ratio (NLR) and absolute neutrophil count (ANC) in patients treated with ziltivekimab, suggesting a direct link between the myeloid cell compartment and systemic inflammation [59].

The ZEUS trial is a large phase 3 trial currently in the recruitment phase. It aims to explore the efficacy of ziltivekimab, administered subcutaneously once a month for 4 years, in reducing MACE in patients with chronic kidney disease (CKD), elevated CRP, and evidence of atherosclerotic cardiovascular disease [60]. Additionally, the recently launched ARTEMIS trial will investigate the efficacy of ziltivekimab in patients admitted with MI to prevent recurrent acute events. The drug will be administered upon admission and once monthly for the following two years [61]. Concurrently, the HERMES trial is testing the efficacy of ziltivekimab in patients with heart failure and systemic inflammation [62].

We eagerly await the results of ongoing trials to add another significant chapter to the relationship between inflammation and atherosclerotic disease.

## Other Therapies

- **ox-LDL Antibody:** Given the pivotal role of oxidized-LDL particles (ox-LDL) in the development of atherosclerotic plaque, they have been evaluated as a therapeutic target in the GLACIER trial, which involved 147 subjects with inflamed carotid or aortic plaques. In these subjects, the administration of a monoclonal antibody against apo-B100 epitopes did not result in significant differences in plaque inflammation, as assessed by 18FDG PET-CT in the index artery, at baseline and 12 weeks follow-up [63]. The main receptor involved in the interaction between Ox-LDL and endothelial cells is LOX-1 (Lectin-like oxidized low-density lipoprotein receptor-1). The phase 2 GOLDILOX-TIMI 69 trial is evaluating the anti-inflammatory potential of a monoclonal antibody targeting LOX-1, and its effect on surrogated markers of atherosclerotic and heart failure events in patients with a history of MI [64].
- **Phospholipase A2 Inhibitors:** Phospholipases A2, both in the lipoprotein-associated form (Lp-PLA2) and the secreted form (sPLA2), catalyze the hydrolysis of phospholipids, generating pro-inflammatory mediators and contributing to the development of atherosclerosis. Elevated circulating levels of Lp-PLA2 and sPLA2 correlate with an increased risk of adverse cardiovascular events [65, 66].
  In the IBIS2 trial, Darapladib, an Lp-PLA2 inhibitor, did not reduce plaque deformability assessed by IVUS nor lower hs-CRP levels in subjects with stable CAD [67]. In two phase-3 trials, one on patients with stable CAD and the other on ACS patients, Darapladib did not reduce MACE [68, 69]. Loss of function gene mutations of PLA2G7, which encode for Lp-PLA2, do not reduce cardiovascular risk, partially explaining the failure of these trials [66].
  Varespladib is a sPLA2 inhibitor. The phase 3 VISTA16 trial with Varespladib was prematurely terminated for futility and a trend toward higher rates of MI [70]; this finding might also be explained by evidence that some sPLA2 isoforms are protective against atherosclerosis [71].

- **5-Lipoxygenase Pathway Inhibitors:** 5-lipoxygenase (5LO) and 5-lipoxygenase activating protein (FLAP) are key enzymes in the production of various inflammatory mediators, such as leukotrienes, from the precursor arachidonic acid. The 5LO-inhibitor atreleuton produced conflicting results. In a phase 2 study involving 191 patients with recent ACS, atreleuton reduced hs-CRP and leukotriene LTB4 levels and slowed the progression of coronary plaques assessed by CT-angiography [72]. Conversely, in a second study of 52 ACS patients, it did not significantly reduce hs-CRP levels nor vascular inflammation assessed by 18FDG PET-CT [73].
  The FLAP inhibitor veliflapon was tested in subjects with MI who had high-risk variants of ALOX5AP gene (encoding FLAP) and LTA4H gene (encoding leukotriene A4 hydrolase). The study showed a dose-dependent reduction in hs-CRP and LTB4 levels [74]. However, genetic studies in mice also have yielded mixed results, suggesting that the 5LO pathway may not play a key role in atherogenesis [75].
  A phase 4 trial is recruiting 200 healthy subjects exposed to air pollution, who will be randomized to receive either the leukotriene receptor antagonist montelukast or a placebo [76]. The trial aims to evaluate the effect of montelukast on endothelial function, carotid intimal media thickness, and systemic inflammation in patients exposed to fine particulate matter (PM2.5, PM10), an emerging cardiovascular risk factor recently correlated with microcirculatory dysfunction [77].

- **MAPK p38 Inhibitors:** p38 mitogen-activated protein kinase (MAPK) is an intracellular kinase activated by various pro-inflammatory stimuli, including ox-LDL, leading to the amplification of the inflammatory response.
  In a study on 99 patients with stable CAD, the MAPK p38 inhibitor losmapimod did not significantly reduce inflammation assessed by 18FGD PET-CT in the index artery [78].
  In the SOLSTICE phase-2 trial losmapimod significantly improved left ventricular ejection fraction and volumes in non-ST elevation MI (NSTEMI) patients, without affecting hs-CRP levels or infarct size [79].
  In the subsequent LATITUDE-TIMI 60 trial, losmapimod reduced hs-CRP and BNP levels in acute MI patients, without reducing the incidence of MACE [80].
  Once again, these conflicting results may be explained by the involvement of p38 MAPK in pro-homeostatic responses, making its inhibition an ineffective therapeutic target [81].

- **NRLP3 Inhibitors:** Inhibition of the NRLP3 inflammasome is attractive for its potential to inhibit both IL-1 and IL-18 production. Some evidence suggest that ventricular dysfunction induced by IL-1 is mediated by IL-18, rather than IL-6, making it a potentially attractive therapeutic target [82]. In a mice model, the NRLP3 inhibitor MCC950 reduced the size and macrophage content of atherosclerotic plaques and decreased the production of inflammatory cytokine, without altering serum lipid levels [83]. TET2 (ten-eleven translocation 2) is a epigenetic modifier enzyme that promote expansion of mutant blood cell. TET2 mutations are associated with clonal hematopoiesis and accelerated atherosclerosis via the NRLP3 inflammasome pathway. Administration of the NRLP3 inhibitor MCC950 in TET2-deficient mice demonstrated to reduce TET2-dependent atherogenesis. These results suggest that inhibiting the NLRP3 inflammasome could mitigate CHIP-related chronic inflammation [84].

- **PTPN22 Inhibitors:** In patients with ACS, plaque destabilization is driven by adaptive immunity disruption, characterized by reduced Treg expansion and increased activation of effector T cells [10]. PTPN22 (protein tyrosine phosphatase non-receptor type 22) is a phosphatase activated by T cell receptor signaling; its overexpression contributes to immunity dysregulation in ACS, making PTPN22 inhibitors promising therapeutic strategies [85].

- **CD31 Pathway**: CD-31, also known as PECAM-1, is an adhesion molecule expressed by several cell types, i.e. platelets and leucocytes, playing a role in cell signaling, activation and potentially involved in plaque disruption. In patients presenting with ACS, the reduction of CD31 in leukocytes is associated with increased inflammation and the exposure at the site of the vascular injury could amplify platelet adhesion. This could promote a further expansion of inflammatory signaling by initiating a homing activity of other cells expressing CD31, such as monocytes and granulocytes, both an unexplored alternative thrombotic pathway, through platelets- CD31 - monocytes aggregates [86]. In the era of tailored medicine, CD31 might represent a promising target in the treatment of inflammatory and thrombotic burden.

- **IL-2 Low-Dose:** IL-2 is an essential cytokine for the activation, proliferation, and regulation of T lymphocytes, including both regulatory T cells and effector T cells. Tregs express high levels of IL-2 receptors (CD25) on their cell surface, making them highly sensitive to low concentrations of IL-2. The protective role of Tregs supports the potential role of low-dose IL-2 as a therapeutic option. In 2021, the phase I-II LILACS trial showed that subcutaneous administration of escalating doses of recombinant IL-2 in 44 patients with stable CAD or NSTEMI resulted in a dose-dependent increase in circulating Treg levels, without significant adverse events [87].The ongoing phase II double-blind, placebo-controlled IVORY trial is investigating the administration of low-dose IL-2 within 14 days of hospital admission in 60 ACS patients, with hs-CRP levels > 2 mg/L. The inflammatory response will be assessed by 18FDG PET-CT of the target vessel before and after low-dose IL-2 administration, while modifications in circulating Treg levels will be the secondary endpoint [88].

## Conclusions

Atherosclerotic cardiovascular diseases remain a significant public health issue, despite advancements in therapies targeting traditional risk factors, such as dyslipidemia, hyperglycemia, and hypertension.

Evidence proved that the paradigm of a single type of culprit coronary plaque as a cause for instability, characterized by a ruptured thin fibrous cap and superimposed thrombosis caused by an inflammatory outburst, does not adequately fit the findings of postmortem and intravascular imaging studies. Indeed, about one-third only of patients with ACS exhibit marked elevation of inflammatory biomarkers associated with widespread coronary, microvascular, and myocardial inflammation and are excellent candidates for anti-inflammatory treatment. In this setting, the immunological mechanisms underlying atherosclerosis represent attractive therapeutic targets to further reduce cardiovascular risk.

The CANTOS trial has been a landmark study, proving for the very first time that anti-inflammatory treatment may reduce cardiovascular risk. Additionally, post-hoc analysis identified a new risk factor for atherosclerosis, i.e. CHIP, highlighting the potential involvement of the hematopoietic system. Other trials, such as the LoDoCo2 and COLCOT trials, provided evidence for cardiovascular risk reduction with colchicine, leading to its inclusion in the 2023 ESC guidelines for patients with recurrent events despite optimal medical therapy according to current guidelines. On the other hand, several trials exploring various molecular pathways, like PLA2 and MAPK p38, did not show clear benefits, possibly due to differences in the role of these pathways between experimental mice models and human atherosclerotic disease.

Implementing anti-inflammatory medications for ACVD is a complex and obstacle-filled journey. To fully exploit the notion that inflammation is a new therapeutic target in the treatment of ischemic heart disease, we need to improve the identification of patients who actually require anti-inflammatory treatment as well as pharmacological targets in inflammatory pathways with a clearly favorable benefit-to-harm ratio. Translational research targeting the inflammatory mechanisms of ACVD faces other several challenges, including the need for large patient cohorts and long follow-up periods, the effective detection of changes in the low-grade chronic inflammation features of atherosclerosis and the necessity of identifying reliable surrogate markers of inflammation. As mentioned, T-cell perturbation may identify patients with ACS at high risk of major coronary events even better than high-sensitivity CRP. A change of focus from soluble markers of inflammation to markers of adaptive immunity dysregulation might be rewarding in the management of patients with ACS.

Further research is needed to explore the potential of immunomodulatory therapies targeting the adaptive immune system, such as low-dose IL-2 and PTPN22 and CD31 pathways modulation.

In the era of precision medicine, single-cell biology and multi-omics approaches may help in appropriate patient selection for clinical trials, identifying new markers of vascular inflammation and evaluating therapeutic responses.

It is time to fully acknowledge the pivotal role of inflammation as a pathogenic driver of CVD and allocate resources towards mechanistic and translational research, to clarify the molecular mechanisms and identify useful therapeutic targets.

## Key References

- Biasucci LM, Pedicino D, Liuzzo G. Promises and challenges of targeting inflammation to treat cardiovascular disease: the post-CANTOS era. Eur Heart J 2020; 41:2164–2167 10.1093/eurheartj/ehz586.
  - CANTOS represented the first step in endorsing anti-inflammatory therapies on top of intense lipid lowering in ACVD. Despite its success, the clinical application of canakinumab faces numerous challenges.

- Byrne RA, Rossello X, Coughlan JJ, et al. 2023 ESC Guidelines for the management of acute coronary syndromes. Eur Heart J. 2023;44(38):3720–3826. 10.1093/eurheartj/ehad191.
  - The latest European guidelines include recommendations for colchicine for patients with uncontrolled risk factors or recurrent events for the first time.

- Engelen SE, Robinson AJB, Zurke YX, Monaco C. Therapeutic strategies targeting inflammation and immunity in atherosclerosis: how to proceed? Nat Rev Cardiol. 2022;19(8):522–542. 10.1038/s41569-021-00668-4.
  - A comprehensive review discussing new therapeutic strategies targeting inflammation and immune response in atherosclerosis.

- Lincoff AM, Brown-Frandsen K, Colhoun HM, Deanfield J, Emerson SS, Esbjerg S, et al. Semaglutide and cardiovascular outcomes in obesity without diabetes. N Engl J Med 2023. 10.1056/NEJMoa2307563.
  - This multicenter, double-blind, randomized, placebo-controlled trial, shows that in patients with preexisting cardiovascular disease and overweight or obesity but without diabetes, exposure to a glucagon-like peptide-1 receptor agonist for ∼3 years reduced the incidence of MACE by 20%.

- Nidorf SM, Fiolet ATL, Mosterd A, et al. Colchicine in Patients with Chronic Coronary Disease. N Engl J Med. 2020;383(19):1838–1847. 10.1056/NEJMoa2021372.
  - This randomized, controlled, double-blind trial helped get colchicine approved as the first drug to target residual inflammation in atherosclerotic cardiovascular disease.

- Ridker PM, Devalaraja M, Baeres FMM, et al. IL-6 inhibition with ziltivekimab in patients at high atherosclerotic risk (RESCUE): a double-blind, randomised, placebo-controlled, phase 2 trial. The Lancet. 2021;397(10289):2060–2069. 10.1016/S0140-6736(21)00520-1.
  - This randomised, double-blind, phase 2 trial shows that ziltivekimab, a fully human monoclonal antibody directed against the IL-6 ligand, safely and effectively reduces biomarkers of inflammation and thrombosis among patients with high cardiovascular risk.

## Data Availability

All data supporting the findings of this study are available within the paper.
